# Encapsulation of Biological Agents in Hydrogels for Therapeutic Applications

**DOI:** 10.3390/gels4030061

**Published:** 2018-07-11

**Authors:** Víctor H. Pérez-Luna, Orfil González-Reynoso

**Affiliations:** 1 Department of Chemical and Biological Engineering, Illinois Institute of Technology, 10 West 33rd Street, Chicago, IL 60616, USA; 2 Departamento de Ingeniería Química, Universidad de Guadalajara, Blvd. Gral. Marcelino García Barragán # 1451, Guadalajara, Jalisco C.P. 44430, Mexico; orfil.gonzalez@cucei.udg.mx

**Keywords:** hydrogel, controlled release, encapsulation, biocompatible, cell therapy

## Abstract

Hydrogels are materials specially suited for encapsulation of biological elements. Their large water content provides an environment compatible with most biological molecules. Their crosslinked nature also provides an ideal material for the protection of encapsulated biological elements against degradation and/or immune recognition. This makes them attractive not only for controlled drug delivery of proteins, but they can also be used to encapsulate cells that can have therapeutic applications. Thus, hydrogels can be used to create systems that will deliver required therapies in a controlled manner by either encapsulation of proteins or even cells that produce molecules that will be released from these systems. Here, an overview of hydrogel encapsulation strategies of biological elements ranging from molecules to cells is discussed, with special emphasis on therapeutic applications.

## 1. Introduction

Polymeric hydrogels are materials consisting of crosslinked polymer chains with high affinity for water. Their crosslinked nature confers them with adequate mechanical properties for a variety of technological applications [1,2]. Hydrogels can be engineered to retain very large water contents while retaining their shape. In fact, it is not unusual to find hydrogels containing more than 99% water [2]. Their large water content was proposed, very early, to result in small interfacial tensions in aqueous environments that would make them highly biocompatible in a variety of biomedical applications [3]. In particular, hydrogels are especially useful for the encapsulation of biological agents because they provide the natural aqueous environment required for biomolecules to function in biological systems. Hydrogels are ideal not only for the encapsulation of proteins and other biomolecules, but also for the encapsulation of living cells. Inherent advantages of hydrogels for these applications are (1) the aqueous environment helps maintain the biological function of the encapsulated material; (2) the crosslinked nature of hydrogels provide a diffusion barrier capable of allowing the passage of molecules of a given size threshold while excluding larger molecules from interacting with the encapsulated elements; (3) the size threshold can be tailored according to the degree of crosslinking of the hydrogel; (4) hydrogels can be created in situ under mild reaction conditions (e.g., temperature and pH), which make them convenient for minimally invasive surgery; (5) hydrogel properties can be selected according to specific applications (e.g., stimuli responsive, degradable, highly or lightly crosslinked, etcetera); (6) hydrogels can be functionalized with biological molecules in order to provide the required biochemical cues for the encapsulated cells (e.g., extracellular matrix proteins or peptides or degradation sequences); and (7) they can provide adequate 3-D microstructure that can modulate the mechano-biochemical transduction signals of encapsulated cells.

**Figure 1:**
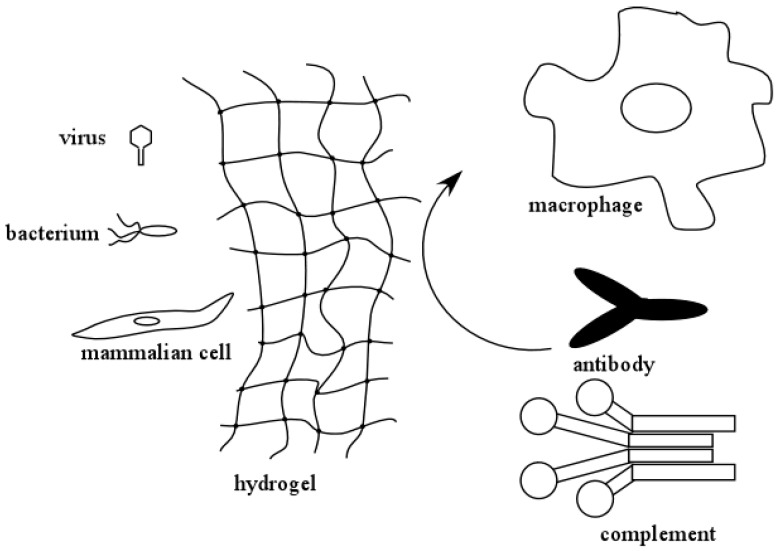
Hydrogels can present barriers that prevent recognition of encapsulated viruses, bacteria, or cells by the immune system. The hydrogel can also provide an internal environment that allows the encapsulated cells to survive changes in environment.

An important feature of hydrogels used to encapsulate molecules or cells is that the crosslinked hydrogel layer can protect the encapsulated biological agents from degradation and/or immune rejection. Figure 1 illustrates this concept. In an encapsulated system, small molecules and solutes can freely permeate and diffuse through the hydrogel layer. However, larger molecules, such as antibodies and components of the complement system, can be excluded from interacting with the encapsulated biological agents. This protects them from immune surveillance and clearance by the humoral (complement system and antibodies) and the cellular (macrophages, B- and T-cells, etc.) immune responses. As a result of this, hydrogels can be conveniently used in medical applications where the therapeutic molecules are being produced by encapsulated cells. The size exclusion capabilities of hydrogels also help protect the encapsulated biological agents against degradation by proteases. The latter is another convenient feature that makes hydrogels attractive for controlled released of biomolecules.

## 2. Hydrogel Preparation

Different preparation methods exist to create hydrogel materials and the polymeric chain can be designed to provide useful properties to these materials. Hydrogels can be formed as homopolymers, copolymers, or multiple polymers. The choice of the hydrogel precursor and/or composition is often made according to the required application. This can include requirements such as biocompatibility, mechanical and transport properties, degradation versus chemical stability, the ability to respond to changes in the environment, and the nature of crosslinks (e.g., covalent, ionic, hydrogen bonds, hydrophobic interactions, and others). The nature of the crosslink interaction in hydrogels is an important factor in their performance [4]. In the drug delivery of biomolecules, it is often desirable to release encapsulated biomolecules over time. This can be accomplished through degradable crosslinks, or by implementing crosslinks that slowly change over time. In other applications, like those involving transplanted cells, it is often desirable to have hydrogel systems that remain stable over time, in order to prevent immune recognition of the encapsulated cells.

The type of crosslinks in hydrogels can have an influence in the properties of hydrogels [4]. By far, the majority of hydrogels are crosslinked by means of covalent interactions. This is because of the ease by which multifunctional monomers can be added in the synthesis process, which often times involves free radical polymerization. However, once they are formed, covalent bonds will not break unless they form part of a degradable moiety. Also, the degradation of the moieties is irreversible, and this can limit the extent to which hydrogel properties can be controlled. In addition to covalent bonds, other types of intermolecular interactions can confer unique characteristics to hydrogels, and non-covalent crosslinks often times can provide hydrogels with unique stimuli responsive or mechanical properties [4]. For example, hydrogen bond crosslinks in hydrogels have been exploited in order to create hydrogels with large elongation and mechanical toughness [5]. In other instances, a combination of ionic and hydrogen bond interactions have been exploited to achieve self-healing properties as well as unique mechanical properties [4,5,6,7]. Crosslinks can also be based on van der Waals and hydrophobic interactions, in order to create hydrogel systems. Approaches used to make hydrogels based on hydrophobic crosslinks often involved amphiphilic ABA triblock copolymers. For example, cholesterol functionalized carbonates with a central hydrophilic block were shown to assemble into gels that could encapsulate drug-loaded polymeric micelles [8]. Poly(ethylene glycol) end capped at both ends with hydrophobic dipeptides, such as tyrosine, have been shown to assemble into hydrogels and have a gel transition close to body temperature [9]. Fluoralkyl-ended poly(ethylene glycol) polymers also assemble into hydrogels at body temperature, and they were exploited for the release of human growth hormone [10,11,12]. Ionic interactions have also been exploited in order to create crosslinks in hydrogels, and sometimes, the properties of hydrogels are conferred by a combination of different intermolecular interactions. In a recent publication, the combination of hydrogen bonds and hydrophobic interactions were exploited in order to create hydrogels with high toughness [13]. As such, the implementation of various types of intermolecular interactions in hydrogels can often be exploited to provide unique properties to these systems. Nonetheless, the flexibility for introducing crosslinks in hydrogels by means of different interactions, such as covalent bonds, hydrogen bonds, ionic and van der Waals interactions, allow encapsulation of biological agents in many different formulations according to the intended application, manufacturing method, and other characteristics of the encapsulated system.

Formation of crosslinks among polymeric chains that have large affinity for water is fundamental for the preparation of hydrogels. Thus, a large number of hydrogel preparation methods involve free radical polymerization. In free radical polymerization, an initiator is decomposed by a variety of mechanisms in such a way that it generates free radicals. These radicals react with the unsaturated double bonds or pi electrons of a monomer in such a manner that they are added to the molecule and transfer the free radical to the monomer. This monomer itself becomes a free radical capable of interacting with more monomers, thus creating a mechanism for chain-growth polymerization [2,14,15]. In order to form crosslinked networks, multifunctional monomers are needed for the formation of crosslinks. Hydrogels intended for biomedical applications can be formed using free radicals chemistry using water-soluble initiators through bulk polymerization [1,16,17,18] or interfacial polymerization [19,20,21,22,23,24,25,26,27,28,29,30]. Furthermore, the decomposition of the initiator can proceed by thermal decomposition, photolysis, or redox reactions [1,2,3,16,17,18]. Free radicals can also be formed using ionizing radiation, electrochemical decomposition, plasma-generated free radicals, and even sonication [2,3,14,16,31]. By far, the use of water-soluble initiators at moderate temperatures using a sensitizer, decomposition of potassium persulfate, or photolytically induced polymerizations, are the preferred methods in applications involving encapsulation of biological elements, due to the fact that it allows the reaction to proceed at mild conditions of temperature and pH.

Other polymerization methods have been used in the formation of hydrogels. Living free radical polymerization methods have been employed to make novel hydrogel materials or precursors. The two main methods are reversible addition fragmentation chain transfer (RAFT) [32,33,34,35] and atom transfer radical polymerization (ATRP) [36,37,38,39,40,41,42,43,44,45]. These methods have been particularly important and useful for the creation of novel hydrogel systems because they allow polymerization to proceed without chain termination by chain transfer. As a result of this, they have been widely used to create polymer brushes and interfaces functionalized with polymers [43,45,46,47,48,49], and to produce block copolymers capable of self-assembling into hydrogel systems [50,51,52,53,54,55,56,57,58,59,60,61]. Additional advantages of these systems are that the block copolymers can be chosen in such manner that they assemble into hydrogels in response to changes in their environment (e.g., pH or temperature).

Other methods exist for the formation of hydrogels, and the mechanisms can involve different types of intermolecular interactions, such as hydrogen bonding, ionic, acid–base, and van der Waals interactions, or a combination of two or more of these interactions [4,5,6,7,8,9,10,11,12,13]. In order to further advance the field, a more complete understanding of the complex interplay of molecular structures and intermolecular interactions will be necessary to further enhance the repertoire of “smart” materials for drug delivery.

## 3. Encapsulation within Hydrogels for Medical Applications

Hydrogels are useful materials in medical applications because of their biocompatibility and high water content [3,62]. Biological molecules can also be incorporated within these systems, in order to guide the desired biological responses [24,63,64,65,66,67,68,69,70,71,72,73,74,75,76]. Not surprisingly, it has been said that hydrogels were the first biomaterials that were *rationally* designed for medical applications [77]. The fact that the composition of the polymeric chains and the nature of the crosslinks can be manipulated independently, allow for wide variations in their properties according to specific applications. Hydrogel crosslinks can be engineered to exhibit properties which makes it possible for a hydrogel to degrade with time. In this regard, hydrogel crosslinks can be designed so they degrade by hydrolysis [78,79,80,81,82,83,84,85,86,87,88,89,90,91,92,93,94,95], or by enzymatic action [64,69,73,75,76,96,97,98,99,100,101,102,103,104,105]. An examination of the scientific literature shows how versatile hydrogels can be in many biomedical applications. The fact that hydrogels can be synthesized under mild conditions of pH and temperature in aqueous environments makes them highly suitable for minimally invasive applications [10,22,24,64,91,106,107,108,109,110,111,112,113,114,115]. It is possible for hydrogels to form, in situ, through photopolymerization of water-soluble monomers and crosslinkers, as hydrogel precursors under physiological conditions of pH and temperature [20,21,22,27,28,67,69,86,110,116,117,118,119,120,121], or by the self-assembly of macromers [6,8,9,10,12,21,50,77,122,123]. It is also possible to form lightly crosslinked hydrogels based on stimuli responsive polymers (e.g., thermoresponsive polymers) that incorporate biomolecules or even cells that can be delivered through minimally invasive procedures. When such materials reach body temperature, the thermoresponsive moieties change the properties of the material from a mostly viscous gel to a solid for localized drug delivery or cell therapies [24,50,73,77,93,97,112,124,125,126,127,128,129]. All these characteristics make hydrogels ideally suited for the delivery of biological agents, ranging from simple biomolecules to entire cells and cell clusters. Thus, they can easily be implemented not only for the delivery of encapsulated drugs, but also to deliver cells and/or when the molecule(s) to be delivered is (are) encapsulated within the hydrogel environment. However, during the formation of hydrogels in vivo, the equilibrium swelling of the material needs to be taken into account. Often times, the swelling of the hydrogel as synthesized may not correspond to its equilibrium swelling. In those situations, the volume occupied by the hydrogel upon formation can change with time. This could have important implications in situations where hydrogels are used in reconstructive surgery (e.g., hydrogel materials containing cells and molecules that are intended to fill a defect in tissue). In those situations, it is important to understand and predict the expected characteristics of the formed hydrogels, so that the formulation of hydrogels precursors can lead, as close as possible, to the formation of hydrogels in a state that is as close as possible to the equilibrium swelling. This can be particularly difficult in the case of stimuli-responsive hydrogels or when the crosslinks are intended to slowly degrade with time. In those situations, it is important to understand the potential effects that the varying volume and/or mesh size of the hydrogel can have in a particular application.

## 4. Proteins and Biomolecules

**Figure 2:**
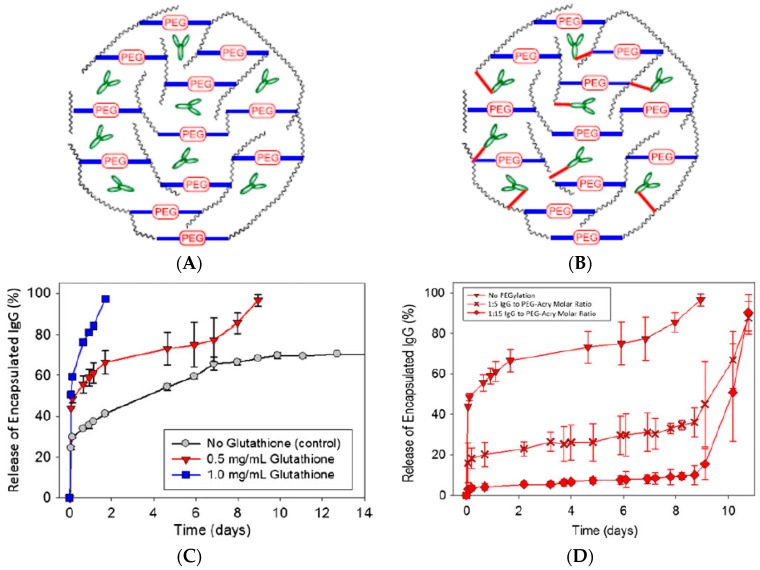
IgG molecules entrapped within a degradable hydrogel. (**A**) The IgG molecules are encapsulated by means of physical entrapment. (**B**) Entrapped IgG molecules are covalently linked to the hydrogel. (**C**) Release of encapsulated IgG molecules (physically entrapped) versus time. Burst release (of IgG in this case) occurs when biomolecules are encapsulated within hydrogels (**A**). (**D**) Release of encapsulated IgG molecules that were covalently linked to the hydrogel. The burst release was delayed when the antibodies were linked to the hydrogel by means of covalent bonds using bifunctional PEG molecules (**B**). Adapted and reproduced with permission from [129].

The fact that hydrogels are cytocompatible and preserve the complex structure and function of biomolecules makes them ideal materials for drug delivery of encapsulated molecules. Many examples abound in the literature on the use of hydrogels for the encapsulation and controlled release of proteins for therapeutic applications. Among the possible proteins to be released from hydrogels, the delivery of growth factors has been widely reported as a strategy to improve wound healing [64,76,130,131,132,133,134,135,136,137,138,139,140,141], release of bone morphogenetic protein 2 (BMP-2) has been used for bone regeneration [142,143,144,145,146,147,148,149,150,151,152], encapsulation of insulin has been explored for oral delivery and/or triggered release under various conditions [153,154,155,156,157,158,159,160,161,162,163,164,165,166,167,168,169,170,171,172], encapsulation of different antigens in hydrogels has been explored in order to enhance the immune response for vaccine boosting effects and other immunotherapies [173,174,175,176,177,178,179,180], and encapsulation of antibodies has been use in localized delivery applications [181,182,183,184]. These are just a few of the many applications that have been explored involving the encapsulation of biomolecules, but they serve to exemplify why hydrogels are such versatile materials for applications involving the encapsulation and release of biological macromolecules. Many times, a burst release is observed in these and other drug delivery systems. In the case of proteins and other macromolecules encapsulated within hydrogels, the burst release can be ascribed to proteins weakly bound to the hydrogel and/or proteins that are encapsulated just within the periphery of the hydrogel. In a recent paper on the encapsulation of antibodies specific for vascular endothelial growth factor (VEGF), we showed that the burst release could go from as little to 25% of the total amount encapsulated to almost 50%, depending on the composition of the hydrogel [129]. Thus, methods to decrease this burst release could involve changing the hydrogel composition. In the same paper, the approach to delay the burst release consisted in covalently linking the antibodies directly to the hydrogel using a bifunctional PEG [129]. These ideas are illustrated in Figure 2. It can be seen that the covalent linkage does not prevent the burst release, but it can delay it by almost 9 days. In this case, the reason was that the linkage did not degrade faster than the hydrogel. Thus, the release occurred once the hydrogel degraded to a significant extent. However, this illustrates that pulsed delivery could be achieved if the rates of degradation of the hydrogel crosslinkers and the molecule linking the antibodies to the hydrogel are carefully chosen.

### Controlled Release of Encapsulated Molecules from Hydrogels

Understanding the release of encapsulated molecules from hydrogels is important in drug delivery applications. Depending on the characteristics of the system, release can occur by a combination of mechanisms that need to be identified in order to better design drug delivery systems based on these materials. One of the most widely used models to analyze drug release involves the use of the Higuchi equation [185] which, in relative amounts released from a drug delivery system, can be expressed as

(1) $$\frac{M_{t}}{M_{\infty }}=k_{H}t^{\frac{1}{2}},$$

where $\frac{M_{t}}{M_{\infty }}$ represents the relative or fractional drug release, $k_{H}$ is a kinetic constant that depends on the diffusion coefficient of the drug and dimensions of the drug delivery system, and *t* is the release time. This equation is based on a model of Fickian diffusion, assuming an infinite sink. In general, it can provide adequate estimates of released drugs during the initial stages of the process, as long as release proceeds by diffusion alone. A system that follows Fickian diffusion then would be expected to show a dependence of initial release that is linear with the square root of time. However, other factors can affect the release of the encapsulated drug in such ways that the release can proceed through the contribution of other mechanisms in addition to diffusion. In those cases, it is not uncommon to observe systems where the initial release does not follow a dependence with the square root of time. Ritger and Peppas proposed the following more general equation in order to model and elucidate potential mechanisms of release [186]:

(2) $$\frac{M_{t}}{M_{\infty }}=k_{1}t^{n}.$$

Here, $\frac{M_{t}}{M_{\infty }}$ represents the relative or fractional drug release, $k_{1}$ is a kinetic constant, *t* is the release time, and *n* has been termed a “diffusional exponent” that provides insights as to the release mechanism from hydrogels. For a thin hydrogel, if *n* = 0.5 the equation becomes similar to Higuchi’s equation, and indicates Fickian diffusion as the release mechanism. When *n* approaches a value of 1, the mechanism can be assumed to be the so called Case II transport, which corresponds to zero order release (the rate of release is constant). This can occur because of concomitant swelling and/or dissolution of the drug delivery system during release. If 0.5 < *n* < 1, the release mechanism can be a complex process involving various mechanisms (e.g., diffusion, dissolution and/or swelling together).

The equations above are normally limited to the initial release stages, but are useful to identify drug release behaviors. For longer release times, the distribution of the drug within the hydrogel changes and decreases with time, leading to slowing of the release rates. An analysis of the release process can be complex when the properties of the hydrogel also change with time (e.g., because of swelling and/or degradation). In the case of diffusion-controlled release, the process has been modeled using Fick’s equation, in which case, the solution depends on boundary conditions and geometry of the system. For initial homogeneous distribution of the drug within the hydrogel and release into a very large system, analytical solutions have been obtained for the following geometries with drugs of a constant diffusion coefficient, *D*, within the hydrogel [187];

Thin film with negligible “edge” effects

(3) $$\frac{M_{t}}{M_{\infty }}=1-\frac{8}{\pi^{2}}\overset{\infty }{\underset{n=0}{\sum }}\frac{1}{{\left(2n+1\right)}^{2}}\exp\left(-\frac{D{\left(2n+1\right)}^{2}\pi^{2}}{L^{2}}t\right)$$

*L*: thickness of the film.

Spherical hydrogel

(4) $$\frac{M_{t}}{M_{\infty }}=1-\frac{6}{\pi^{2}}\overset{\infty }{\underset{n=0}{\sum }}\frac{1}{n^{2}}\exp\left(-\frac{Dn^{2}\pi^{2}}{R^{2}}t\right)$$

*R*: sphere radius.

Cylindrical hydrogel device

(5) $$\frac{M_{t}}{M_{\infty }}=1-\frac{32}{\pi^{2}}\overset{\infty }{\underset{n=1}{\sum }}\frac{1}{q_{n}{}^{2}}\exp\left(-\frac{q_{n}^{2}D}{R^{2}}t\right)\overset{\infty }{\underset{p=0}{\sum }}\frac{1}{{\left(2p+1\right)}^{2}}\exp\left(-\frac{D{\left(2p+1\right)}^{2}\pi^{2}}{H^{2}}t\right)$$

*q_n_*: are the *n*th roots of the Bessel function of the first kind of zero order; *R*: cylinder radius; *H*: cylinder length.

Since models that predict release of drugs encapsulated within hydrogels are mainly based on the assumption of a diffusion mechanism, they require knowledge of diffusion coefficients within hydrogels. Since normally only the bulk diffusion coefficients are available, it is necessary to account for the effects that hydrogels can have in the diffusion coefficients of the encapsulated molecules. For example, if the size of the released molecules was much smaller than the mesh size of the hydrogels, the use of bulk diffusion coefficients could provide adequate predictions of the drug release process. However, in the case of release of macromolecules such as proteins from hydrogels, the size of these molecules can be comparable to the mesh or “pore size” of the hydrogel. In those cases, the diffusion coefficients could differ significantly from bulk diffusion coefficients. As a result of this, several models have been developed that attempt to model the effects that hydrogel chains have on the diffusion coefficients of macromolecules. Existing models are based on different mechanisms invoked to explain such effects, namely: free volume theory [188,189], hydrodynamic theory [189,190], or obstruction theory [189,191,192,193]. These theories have led to the development of different equations that attempt to describe how the hydrogel environment affects diffusion coefficients. Such models are able to represent the experimental data on diffusion of molecules within hydrogels to different extents. In other words, the models can predict diffusion coefficients in some polymer systems better than others, which indicate limitations in our knowledge of all the hydrogel characteristics that influence diffusion of molecules. Comparisons between models have been described elsewhere [189,192]. In what follows, only some representative models for the different theories are given.

In free volume theory, diffusion is described to occur when the solute “jumps” into voids formed in the solvent space by redistribution of the free volume within the liquid [189,192]. Solute diffusion depends on the jumping distance, thermal velocity of solute, and the probability that there is a hole free volume adjacent to the solute molecule. The hydrodynamic theory describes solute transport in terms of the Stokes–Einstein equation, whereby the solute is modeled as a hard sphere that is large compared to the space of solvent in which it moves, and the solvent is treated as a continuum [190]. In hydrodynamic theory models, the frictional drag on the solute is affected by the polymer chains, which enhance frictional drag by slowing down the solvent near the polymer chains. Thus, hydrodynamic theory models focus on modeling the friction drag coefficient [189,192]. Obstruction theory models assume that the polymer chains in the hydrogel cause an increase in the pathlength for diffusion. The polymer chains can be conceptualized as a sieve that allows the passage of molecules only if they can pass between the polymer chains.

The following are some equations derived from the different theories. It is not an extensive list of the different equations developed to predict diffusion coefficients within hydrogels, but they are given as illustrative examples. In these equations *D_g_* is the diffusion coefficient of a molecule within the hydrogel, *D*_0_ is the bulk diffusion coefficient of the molecule, *r_S_* is the radius of the solute, *r_f_* is the radius of the polymer fiber, and φ is the volume fraction of the polymer in the gel.

Free volume theory model by Lustig and Peppas [194]

(6) $$\frac{D_{g}}{D_{0}}=\left(1-k_{1}r_{S}\varphi^{0.75}\right)\exp\left(-k_{2}r_{S}^{2}\left(\frac{\varphi }{1-\varphi }\right)\right)$$

In this equation, *k*_1_ and *k*_2_ are treated as fitting parameters.

Hydrodynamic theory model by Cukier [195]

(7) $$\frac{D_{g}}{D_{0}}=\exp\left(-k_{C}r_{S}\varphi^{0.75}\right)$$

Here, *k_c_* is an undefined constant for a given polymer–solvent system that must be fitted to the data.

Hydrodynamic theory model by Philips et al. [196]

(8) $$\frac{D_{g}}{D_{0}}={\left[1+{\left(\frac{r_{S}^{2}}{k}\right)}^{\frac{1}{2}}+\frac{1}{3}\frac{r_{S}^{2}}{k}\right]}^{-1},$$

where *k* is the hydraulic permeability of the medium, which can be estimated for hydrogels using the correlation developed by Jackson and James [197]:

(9) $$k=0.31\;r_{f}^{2}\varphi^{-1.17}.$$

Obstruction model by Ogston et al. [198]

(10) $$\frac{D_{g}}{D_{0}}=\exp\left[-\frac{r_{S}+r_{f}}{r_{f}}\sqrt{\varphi }\right]$$

Obstruction model by Amsden [193]

(11) $$\frac{D_{g}}{D_{0}}=\exp\left[-\pi {\left(\frac{r_{S}+r_{f}}{k_{S}\varphi^{-1/2}+r_{f}}\right)}^{2}\right]$$

An analysis of the effectiveness of these equations in predicting solute diffusion within hydrogels can be found elsewhere [189,192]. These equations also illustrate the complexities of modeling drug release from hydrogels, especially in situations where the swelling changes as a function of time, due to crosslink degradation, changes in activity coefficients of the systems with changing solute concentrations, and changes in swelling due to non-equilibrium or changing equilibrium conditions.

## 5. Viruses

Although viruses are normally thought as pathogenic biological entities causing a multitude of diseases, they can also be employed in some applications with potentially beneficial health effects. Two potential applications of these systems are as an alternative to antibiotics (bacteriophages) and as vectors for delivery of nucleic acids in genetic engineering applications. However, their use in therapeutic applications is often times limited because of immune clearance, inactivation by natural defenses or barriers in the body, and the need to direct them to specific sites in the body. In this regard, hydrogel materials can help circumvent some of these limitations. Similar to other applications, encapsulation of viruses within hydrogels offers protection against immune recognition because the hydrogel material surrounding the encapsulated viruses offers a physical barrier against the many elements of the immune system.

For some applications of phage therapy, oral administration is a convenient therapeutic method. However, in order for such a route of administration to be effective, two major requirements need to be addressed. One is avoiding the harsh acidic environment of the stomach, where the low pH can quickly inactivate the phages administered via oral ingestion. For this, it may be necessary to encapsulate the phages in a system that offers protection against acidic inactivation. The other is the need to release the encapsulated phages after passage through the stomach (e.g., in the small intestine). These requirements can be met with a hydrogel system that isolates encapsulated phages from the low pH environment and, subsequently, releases the phages from the hydrogel when the pH increases in the small intestine.

Oral administration of bacteriophages has been proposed as an alternative to the use of antibiotics for controlling the presence of enterohemorrhagic *E. coli* in cattle [199,200]. This is an important application, since enterohemorrhagic *E. coli* is the main cause of hemolytic uremic syndrome, and cattle are the main reservoir of the pathogenic forms of *E. coli* causing this disease [201]. This approach is also attractive as an alternative to the indiscriminate use of antibiotics, which can result in the emergence of antibiotic resistant bacteria. As mentioned, oral delivery of bacteriophages for controlling these bacteria presents the challenge of the need to bypass the harsh acidic conditions of the stomach, which could quickly deactivate the bacteriophages before they reach the intestine, where enterohemorrhagic *E. coli* reside. Also, it is necessary for any delivery system to respond to the conditions of the intestine, such that bacteriophages are quickly released in order to control the pathogenic bacteria. Phage CA933P is capable of infecting enterohemorrhagic strains of *E. coli*, such as O157:H7, O145:H25, O13:H8, and ONT:H12, as well as other Gram-negative bacteria. However, it is quite sensitive to pH values below 4, which precludes direct oral administration [202]. For this reason, phage CA993P was encapsulated within alginate and low methoxylated pectin materials, in order to see their suitability for oral delivery to the intestine [199,200]. Hydrogel microspheres containing the encapsulated phages were formed by ionic crosslinking of these polysaccharide materials in the presence of calcium ions. The microspheres were made using the corresponding polymer solutions or using the polymer solutions emulsified with oleic acid [199,200]. It was reported that emulsified low methoxylated pectin was more efficient as a delivery system than unemulsified pectin, alginate, or emulsified alginate microspheres. Exposure of free (non-encapsulated phages) to pH 2.5 alone resulted in the reduction of bacteriophage titer by 3.7 logarithmic orders after only 10 min. Furthermore, at pH 2.5 and in the presence of 0.5 mg/mL pepsin, the phage titer decreased to undetectable levels within only 10 min. On the other hand, when bacteriophage CA993P was encapsulated within emulsified low methoxylated pectin spheres, there was no significant decrease in phage titer at pH 2.5 and concentrations of pepsin up to 4.2 mg/mL after 3 h [199]. Release of phages proceeded almost to completion after 4 h in phosphate buffer solution (PBS) at pH 7.2 and 37 °C [199,200], which are conditions of pH and temperature similar to what would be expected in the intestine. These are two important factors for the intended application, namely, that the encapsulated phages are protected from the harsh environment of the stomach, and that they can be quickly released under the conditions of the intestine. The release was also found to follow the Korsmeyer–Peppas model, which includes swelling, diffusion, and dissolution of the polymeric matrix [200].

**Figure 3:**
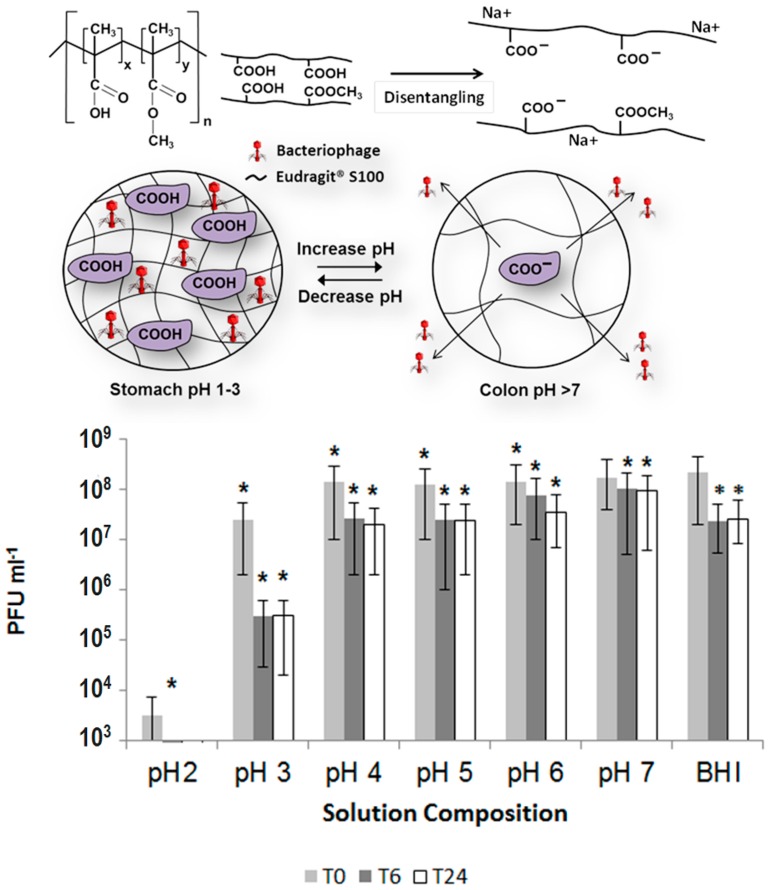
(**Top**) Concept of encapsulation of bacteriophages within an ionic hydrogel. At low pH, the carboxylate groups remain protonated, and the hydrogel does not swell with the acidic fluids of the stomach. Once the particles reach the small intestine, the change in pH causes the carboxyl groups to become deprotonated (hence changed and more polar), which causes the hydrogel to swell and release the encapsulated bacteriophages. (**Bottom**) Number of plaque forming units from a system with encapsulated bacteriophages after 0 h (T0), 6 h (T6), and 24 h (T24) of residence within the hydrogel particles at the given pH values. (* indicates significantly different phage titres using a 2 sample *t*-test at each condition compared with phage at T0 for each composition (*p* < 0.05).). (Adapted and reproduced with permission from [206].)

Encapsulation of bacteriophages offers significant promise to address some medical problems. *Clostridium difficile* often causes intestinal infections that are especially difficult to treat [203] and the treatment can come at significant costs [204]. Treatment of *C. difficile* is particularly difficult with antibiotics, and recurrent infections often times occur [205]. Thus, delivery of bacteriophages is a promising treatment for this condition. The *C. difficile* specific bacteriophage, *myovirus CDKM9* was encapsulated within the commercial pH responsive polymer Eudragit^®^ S100 using a microfluidics system as proof of concept of the potential for treatment of *C. difficile* [206]. The bacteriophages were encapsulated within monodisperse microspheres about 100 μm in diameter. The encapsulated phages remained stable and viable for 4 weeks at 4 °C. There was no significant release of phages under simulated gastric conditions at pH 2 for 3 h. However, the microsphere showed significant release of active phages for 24 h at pH 7, which offers the potential of this technology for the treatment of *C. difficile*. The concept of encapsulating *C. difficile*-specific phages for delivery to the intestine is shown in Figure 3. Here, the polymer contains carboxyl groups that remain protonated within the acidic environment of the stomach. This allows for protection of the encapsulated phages because the hydrogel remains collapsed and does not swell with the acidic fluids of the stomach. Once these particles reach the small intestine, the pH change causes the carboxylic groups to deprotonate, which causes the hydrogel to suddenly swell because of electrostatic repulsion and the more polar carboxylate groups, which causes the encapsulated bacteriophages to be released in the small intestine.

In another further medical application, encapsulation of bacteriophages has been explored in order to address the problem caused by the formation of biofilms of *Proteus mirabilis* on urinary catheters. *Proteus mirabilis* can cause significant complications in catheter-associated urinary tract infections because it produces bacterial urease enzymes that generate ammonia upon reaction with urea, thus increasing the pH of urine. This can result in local supersaturation and precipitation of struvite and apatite, causing their accumulation in the catheter walls, and eventually leading to obstruction of the catheter [207,208,209,210]. In order to address this potential problem, a coating for catheters was designed consisting of a hydrogel layer containing encapsulated bacteriophages on the surface of the catheter. This layer was covered with the pH-responsive polymer EUDRAGIT^®^S 100 [210]. This coating showed that, using an in vitro bladder system, catheter blockage was doubled under conditions of *P. mirabilis* infection. Also, under such conditions of catheter infection, a “burst release” of the encapsulated phages occurred in response to the rapid local pH [210]. Thus, this approach also shows the potential of phage encapsulation for medical applications.

Even for tissue engineering applications, the encapsulation of viruses can offer useful applications. Encapsulation of lentiviruses within degradable poly(ethylene glycol)-based hydrogels has been proposed as a method to enhance transgene expression [211]. By increasing the residence time within the hydrogels, it was shown that prolonged and localized gene expression of pLenti-CMV-GFP and pLenti-CMV-GLuc could be achieved [211].

In some applications, it is also necessary to study the long-term protection effects of encapsulated bacteriophages. The *Salmonella enteritidis* specific phage f3αSE, when encapsulated within alginate spheres crosslinked with Ca^2+^ ions, was shown to remain viable at low pH conditions and higher temperatures for extended periods of time [212]. In fact, alginate-encapsulated bacteriophages remained infectious for long periods of time. In a water flow system, the encapsulated phages could be kept at concentrations of 10^2^ to 10^4^ PFU/mL for 250 h, which makes them suitable for oral administration in water feeding systems for poultry.

Other concepts on the release of encapsulated bacteriophages have been explored. The antimicrobial bacteriophage K (ΦK) has been encapsulated within agarose gels upon which a layer of methacrylate modified hyaluronan hydrogel were photocrosslinked [213]. These types of materials would respond to infectious agents such as *Staphylococcus aureus* strains, which are known to release hyaluronidase as an important virulence factor secreted by some pathogenic bacteria. In the presence of hyaluronidase positive pathogenic bacteria, the degradation of the methacrylated hyaluronan hydrogel would result in the release of the encapsulated bacteriophage K, which would subsequently infect the pathogenic bacteria. In this manner, this system was designed as a hydrogel capable of responding to bacterial infection.

## 6. Bacteria

With the increasing understanding of the role of the microbiome in human health, it is becoming evident that there is a need to have methods to efficiently deliver probiotics to the intestine. Oral administration of probiotics, although widely used, cannot be effective without a method of protection against the low pH of the stomach, and the need for quick release at the pH of the intestine. Furthermore, any encapsulation method also needs to maintain the viability of encapsulated microorganisms during storage of the product until administration. Thus, the hydrogel system must offer the capability of quickly releasing the encapsulated bacteria in the intestine in order to allow colonization of the intestinal tract with beneficial bacteria [214,215,216,217].

**Figure 4:**
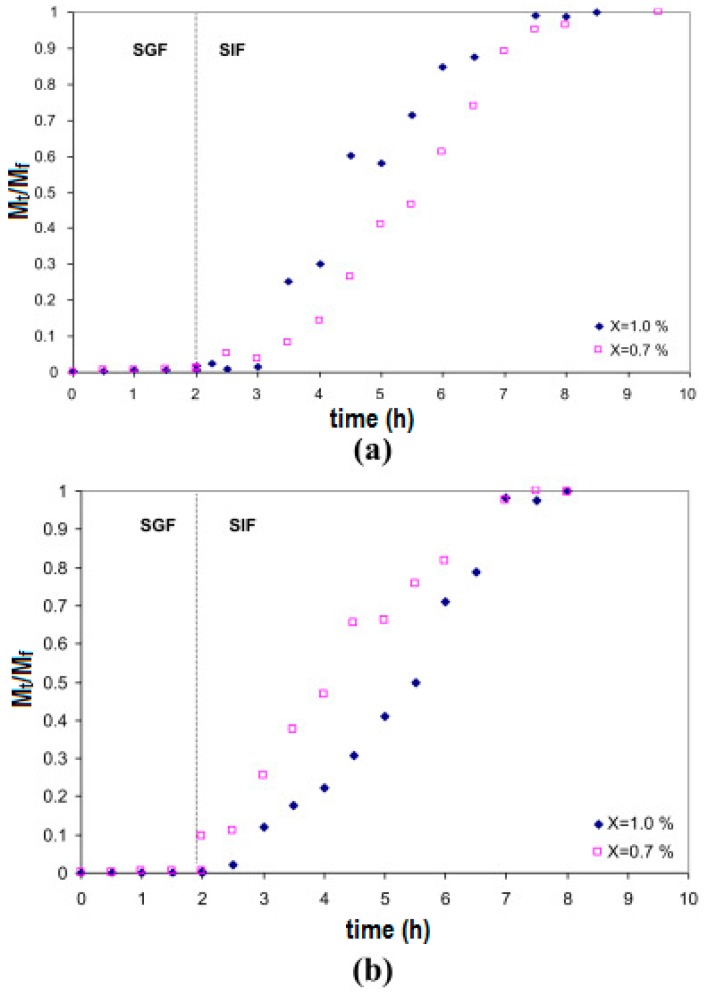
Fractional release of *P. acidilactici* from xanthan–chitosan capsules prepared under four different conditions. The release pH conditions are pH = 2.0 for SGF and pH = 6.8 for SIF. (**a**) Chitosan: 0.7% (*w*/*v*), pH = 6.2; Xanthan: 0.7% (*w*/*v*) and 1.0% (*w*/*v*); (**b**) Chitosan: 0.7% (*w*/*v*), pH = 4.5; Xanthan: 0.7% (*w*/*v*) and 1.0% (*w*/*v*). (Reproduced and adapted with permission from [218].)

Work on the encapsulation of probiotics for oral delivery to the intestines is emerging in the literature. Different hydrogels have been explored and shown to be able to protect encapsulated bacteria from the low pH environment of the stomach, while allowing quick release at the pH conditions of the intestine. Xanthan–chitosan hydrogels exhibited negligible release of encapsulated *Pediococcus acidilactici* in water or simulated gastric fluid at pH 2.0 for two hours [218]. The pH of simulated gastric fluid seemed more critical than the presence of pepsin in the release of encapsulated bacteria. The encapsulated bacteria were completely released in simulated intestinal fluid in 5 h. Furthermore, the xanthan–chitosan capsules were stable in water for at least 3 days, and encapsulation within these hydrogels showed six-log retention of viability over non-encapsulated *P. acidilactici* for 1 h at pH 2.0, and four-log retention of viability at pH 2.0 after 4 h [218]. Figure 4 shows that the encapsulated bacteria remain within the hydrogel capsules under low pH conditions (simulated gastric fluid or SGF) for at least two hours, and that when the pH is changed to that found in the intestine (simulated intestinal fluid or SIF) swelling of the hydrogel capsules and release of the bacteria occur soon after, and continue for at least 5 h. This shows how hydrogels allow the formulation of release systems, where bacteria are protected from the harsh stomach environment for the typical duration of food passage through the digestive system.

Alginate/pectin was also explored for the encapsulation of *Lactococcus lactis* within beads of these hydrogel materials. *L. lactis* is of importance because it produces and releases nisin, a polycyclic antibacterial peptide. Nisin, in combination with miconazole, has been used in the treatment of *C. difficile*, a bacterial intestinal infection that is particularly difficult to treat [219]. Thus, the encapsulation of *L. lactis* could be considered as another potential application for the controlled release from hydrogels of molecules produced by encapsulated bacteria for therapeutic applications. A 75/25 alginate/pectin ratio was found to be optimum for providing the best mechanical properties of beads containing *L. lactis*, and also allowed for the best release of nisin during the storage period. *Lactobacillus casei* encapsulated within an alginate–pea protein isolate matrix and was found to be protected during freeze drying compared to free cells. Storage in the frozen state was achieved for 84 days, although the freeze-dried cells exhibited a weaker protection against the low pH of simulated gastric acid conditions, but showed similar release profiles in simulated intestinal fluid [220]. *Bifidobacterium longum*, an obligate anaerobe that establishes a protective gut microbiome in infants through adulthood, was encapsulated within alginate microgels that were subsequently coated with chitosan [221]. Interestingly, the chitosan layer did not seem to improve the viability of the encapsulated *B. longum*. In fact, storage and gastrointestinal stability decreased when compared to alginate beads without the chitosan coating, which further indicates the need to more fully understand the complexity of these systems. Another less elegant approach explored was the formation of compressed pellets of lyophilized probiotics (*L. acidophilus*) together with dry sodium alginate. In this case, the pressed cell pellet was carefully surrounded by dry alginate before further compression into pellets. Upon hydration, this system provided probiotics surrounded by the hydrated hydrogel, which also offer protection against acidic challenges and release under simulated intestinal fluid conditions [222].

## 7. Islet Cells

Hydrogels can help isolate cells against immune recognition. Semipermeable hydrogel membranes around cells protect them against immune recognition, while at the same time, they allow the passage of nutrients, oxygen, and small molecules. Because of this, hydrogels are ideal materials for transplantation of cells that offer therapeutic benefits. This is why hydrogel capsules have long been explored for the encapsulation of cells. Probably one of the first applications of encapsulated mammalian cells for therapeutic applications in humans was for the purpose of treating type I diabetes [223]. The first clinical trial of immunoprotected islet cells in humans was published in 1994 [224]. This involved alginate as the encapsulating material for islet allografts implanted in humans. Islet cells were isolated from human cadaver donors by collagenase digestion and gradient separation. Then they were cultured and coated by an alginate–polylysine–alginate membrane, and implanted in the peritoneal cavity of a 38 years old adult patient who had been insulin dependent for 30 years. The procedure was successful in that the patient was able to become insulin independent for 9 months following the procedure. This success followed the earlier work by the same group on islet transplantation in diabetic dogs, which showed they could become insulin independent following transplantation of alginate encapsulated islets for 172 days, and that the implanted islets remained viable for as long as 726 days [225]. Encapsulation of islets within alginate capsules is not without problems. Earlier attempts at using this technology for the treatment of insulin dependent diabetes failed because mannuronic acid monomers from alginate capsules were found to stimulate interleukins 1 and 6, as well as tumor necrosis factor, which resulted in the formation of fibrosis due to fibroblast proliferation around the implanted capsules [225]. Mechanical integrity was also found to be important, and directly influenced by the amount of mannuronic acid in the alginate [225]. For these reasons, alginate high in guluronic acid needed to be used. Also, after formation of alginate capsules around the islets, a layer of polylysine was formed in order to confer better mechanical stability. Since polycations can elicit fibrous capsule formation [226], another layer of alginate needed to be placed around the capsules, which resulted in the alginate–polylysine–alginate coating. This also illustrates the need to better understand the biocompatibility of hydrogel materials in applications where encapsulated cells need to be implanted [223,224,225].

**Figure 5:**
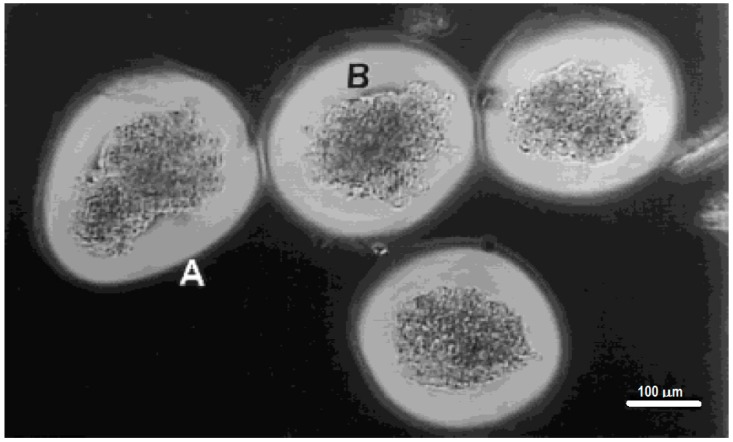
Porcine islet cells encapsulated with a PEG diacrylate-based hydrogel using interfacial photopolymerization. The thin, outermost zone (labeled A) was presumed to be less crosslinked, and the filamentous nature of this outer zone may be due to the incomplete crosslinking of the hydrogel at the termination of laser illumination. Because the photoinitiator was present only at the islet surface, the thicker inner zone, closer to the islets (labeled B) and thus closer to the eosin Y photoinitiator, was presumably more highly crosslinking and thus had a more dense appearance. (Reproduced and adapted with permission from [20].)

A slightly different approach for the encapsulation of islets within hydrogels involves the photopolymerization of poly(ethylene glycol) diacrylate (PEG-DA) around islets [116,117,227]. This system involves the interfacial photopolymerization of PEG-DA around eosin-Y stained islets. The photopolymerization reaction starts at the eosin-Y molecules [28] and produces a layer of crosslinked PEG-DA hydrogel that extends only about a 100 to 300 μm from the interface [28,30,228,229], and can be controlled by varying the polymerization conditions [20,228,229]. The advantages of this approach are various: (1) poly(ethylene glycol) has been found to exhibit excellent biocompatibility in most biomaterial applications; (2) the polymerization conditions maintain cell viability; (3) the thin layers of hydrogel formed around the islets permit efficient transport of oxygen and nutrients to the islets; (4) the crosslinked nature of the hydrogels presents an immunoisolation barrier against components of the immune system, such as antibodies and complement components; (5) the covalent nature of the crosslinks is more stable than divalent cation crosslinked alginate; and (6) the coating is non-ionic (unlike alginate). The last two factors contribute to the biggest advantages compared with alginate capsules in terms of biocompatibility and stability. Encapsulation of islets within photopolymerized PEG-DA was shown to be successful in xenograft transplants of porcine islets in the peritoneal cavity of Sprague-Dawley rats. The encapsulated islets were shown to be glucose responsive. Normoglycemia was maintained in diabetic induced rats for up to 110 days after implantation of 5000 to 8000 encapsulated islets. This shows promise on this approach for the treatment of insulin dependent diabetes [117]. Figure 5 shows the uniformity and conformal coating of PEG diacrylate hydrogels around porcine islet cells [20].

## 8. Tissue Engineering

Encapsulation of cells within hydrogels can serve many applications in medicine. The applications discussed above involved the delivery of cells or molecules produced by the cells and the hydrogel was, for the most part, an inert material. However, there are many applications in which the hydrogel provides more than an encapsulating material, a physical barrier against the immune system, or a simple vehicle for the delivery of cells. The hydrogel itself can constitute the environment that allows cells to differentiate and/or maintain certain phenotype. In this regard, the system that is delivered in the body consists of hydrogel and cells altogether. Thus, the hydrogel itself, along with cells and biochemical molecules, can constitute an active part of the system that is delivered to certain tissues for cell therapy purposes.

Work on cartilage tissue engineering often relies on the use of hydrogels to deliver chondrocytes as well as maintaining their phenotype. An advantage of hydrogels is that they can be created under mild conditions compatible with the viability of cells, such as chondrocytes, and often times, the hydrogel properties are designed in order to allow for minimal invasive surgery applications [87,91,107,109,112,230,231,232,233,234]. Many different types of hydrogels have been studied for the formation of cartilage using encapsulated chondrocytes. Alginate has been shown to induce redifferentiation of chondrocytes that were dedifferentiated by monolayer cell culture [235]. Alginate/hyaluronan composite hydrogels loaded with TGF-beta 1 have also been demonstrated to allow the differentiation of periodontal ligament stem cells and human bone marrow mesenchymal stem cells into a chondrogenic phenotype. Such differentiation of these cells into a chondrogenic phenotype was shown to be dependent on the modulus of elasticity of the hydrogel delivery system [236]. It is likely that the similarity of hydrogel mechanical properties and cartilage allow the cells to be differentiated into a chondrogenic phenotype.

Other hydrogels have also been explored for cartilage regeneration. Thermoresponsive polymers with low critical solution temperatures below 37 °C are promising materials because of their capability to be made into lightly crosslinked hydrogels that can be delivered through a needle into the body. When they reach body temperature, they solidify and remain in place, adopting the shape of the environment they were delivered to. Healy et al. has synthesized poly(*N*-isopropyl acrylamide) based hydrogels containing peptide-based crosslinks that are degraded specifically by metalloproteinases involved in the remodeling of the extracellular matrix during the wound healing reaction [23,112]. Such hydrogels were found to support bovine articular chondrocyte viability for at least 28 days in vitro. They could be injected through a small needle and quickly stabilized in situ by the thermoresponsive properties of the hydrogel materials. The chondrocytes encapsulated in such thermoresponsive hydrogels produced extracellular matrix with properties similar to natural cartilage. They stained for sulfated polysaccharides, and there was evidence that the hydrogel material allowed for the proliferation of chondrocytes within those hydrogels [112]. Thus, thermoresponsive hydrogels hold great promise for minimal invasive applications.

Photopolymerization reactions are fast and can be used to create hydrogels in situ under mild reaction conditions compatible with cell viability. Thus, they also have the potential to be implemented as minimally invasive surgical procedures for cell-based therapies. For this reason, photopolymerized poly(ethylene glycol) based hydrogels have been explored for tissue engineering of cartilage. Polyethyleneoxide dimethacrylate photopolymerized into thick, 2 to 8 mm thick disks of 9 mm diameter, was shown to successfully encapsulate and maintain the viability and differentiation of chondrocytes. The chondrocytes were shown to produce extracellular matrix components characteristic of cartilage tissue. The specimens showed homogeneity in the distribution of cells and extracellular matrix components throughout the sample. Furthermore, they were shown to produce glycosaminoglycan and collagen homogeneously throughout the hydrogels, which is promising as a technology for cartilage repair [237].

Hydrogels have been also implemented in hard tissue applications. Their versatility to be formed in situ under mild conditions, which are compatible with the integrity of biomolecules and cells, made them ideal materials for the treatment of bone defects and to accelerate healing in orthopedic applications [148]. Different hydrogel systems have been explored for bone healing applications. In a good number of these applications, the encapsulation and delivery of growth factors and molecules that induce the stimulation of osteoblasts is employed. The encapsulation and delivery of bone morphogenetic protein 2 (BMP-2) has been widely employed in bone healing/regeneration applications. This molecule has been shown to successfully allow for bone regeneration when incorporated in hydrogels [149]. BMP-2 has been used in PEG [75,151,152,238], alginate [142,144,147,149,236], polyphosphazenes [146], and elastin-like peptides [143]. Some systems combine more than one hydrogel component, and often incorporate a component of the extracellular matrix, such as hyaluronic acid [142], or the introduction of RGD peptide sequences that allow cell adhesion [75,143,148]. The encapsulation and delivery of BMP-2 has also been explored alone or together with other biomolecules, such as platelet-derived growth factor (PDGF) [143,144]. It is noteworthy to indicate the advantage to incorporate molecules such as BMP-2 with hydrogels in bone-healing applications. In a recent paper involving the encapsulation of human bone marrow cells within hydrogels containing alginate/hyaluronic acid and BMP-2, there was an enhanced osteogenic differentiation in vitro and bone regeneration in vivo, compared with the hydrogel alone or BMP-2 [142]. This is an indication that hydrogels play a synergistic role when combined with cells and signaling molecules.

The fact that hydrogels can be compatible with the viability of cells, can preserve the biological function of proteins and other biomolecules, and also can be injectable or polymerized in situ under mild conditions, make them important materials for stem cell-based therapies [70,100,137,142,143,144,152,236,238,239,240,241]. Research on stem cells indicates that their differentiation is dependent on a number of environmental signals, among which the rigidity of the matrix plays an important role [242]. The mechanical properties (hence the rigidity) of hydrogels are strongly dependent on their degree of crosslinking. For this reason, there have been a number of studies that indicate that the mechanical properties of hydrogels, which can be controlled through the degree of crosslinking, can have an effect in the differentiation of stem cells [243,244,245,246]. Many different applications can be found in the literature about the use of hydrogels in stem cells therapy. Injectable hydrogels carrying stem cells have been explored for cardiac tissue repair [247,248]. Injectable hydrogels based on PEG, or Tetronic^®^ conjugated with fibrinogen, were shown to result in improvement of heart function after inducing infarcts in a rodent model through ligation of the left descending coronary artery. The results indicated that the hydrogels with the highest modulus provided greater recovery of heart function and neovascularization. This indicated that hydrogels, by themselves, had a therapeutic effect in treating myocardial damage after an infarct by preventing adverse remodeling of tissue after a heart attack [249]. Thus, it would be reasonable to expect that encapsulated stem cells would further contribute to recovery of damaged myocardium after an infarct. Injectable fibrin gels have been used to deliver bone marrow mononuclear cells to the cryoinjured infarcted myocardium of a rat animal model. Implantation of bone marrow mononuclear cells delivered in a fibrin matrix resulted in significantly higher neovascularization than cells implanted alone [250]. Other approaches have involved bone marrow-derived mononuclear cells in PEG–fibrin hydrogels with covalently bound hepatocyte growth factor, which resulted in cell prevalence increases of 15-fold in hearts receiving the matrix containing the hydrogel, cells, and growth factors, compared to control that received a saline injection only [251]. Other uses of hydrogels in conjunction with stem cells have included stem cells for neural tissue repair [243,252,253,254]. In those applications as well, the hydrogels extend the survival of the stem cells, and can modulate their differentiation. The differentiation is influenced by the mechanical properties of the hydrogel, as well as more complex factors, such as their porosity and whether signaling molecules are also encapsulated in the hydrogel systems.

## 9. Conclusions and Future Directions

Hydrogels are highly versatile materials for uses in many medical applications. There are many characteristics of these systems that make them attractive for biomedical applications. Their high water content is compatible with the biological activity of biomolecules and the viability of cells. Hydrogels offer many possibilities in the delivery of biological systems, such as molecules and cells, that would not be possible with other materials. Many examples can be found in the literature on their use for the encapsulation and delivery of biomolecules, viruses, bacteria, and mammalian cells for medical applications. The fact that hydrogels can be made under mild conditions of temperature and pH make them ideal not only for the encapsulation of the fragile living systems and biomolecules involved, but also permit their formation in situ for minimally invasive applications. Given that other molecules, such as adhesive [24,63,64,67,69,70,71,72,73,74,75,96,106,125,143,255,256,257,258,259] and/or degradation [64,73,76,97,98,99,106,109] sequences, and signaling molecules, such as growth factors [4,68,91,107,130,131,133,134,135,136,137,138,139,140,141,144,148,150,151,152,248,255,260,261,262,263,264,265], can be incorporated into these systems, offers the possibility not only to deliver cells, but also to modulate their function according to the required response. These are just some of the most salient characteristics of hydrogels that makes them useful in medical applications. However, these systems can be made with many other interesting characteristics. Their mechanical properties can be controlled or modulated according to the degree of crosslinking and chemical composition, which is important in many soft tissue applications, but also because the mechanical properties can influence the function and differentiation of cells [12,138,149,243,244,254,266,267]. Some hydrogels can be stimuli-responsive, such that they change properties in response to environmental cues, like temperature, pH, light, electrical stimuli, and others [77,93,268,269,270,271,272]. With the variety of properties that hydrogels can be prepared with, it is no surprise that they are widely used in many medical applications that combine many useful characteristics. It is likely that in the future, more examples with higher levels of sophistication will begin to emerge. For these, it will be necessary to develop a larger variety of stimuli-responsive hydrogels, degradable hydrogels, micro and nanostructured hydrogels, microfabricated hydrogel structures, and self-assembly methods to create supramolecular structures with these materials. Other potential properties would be gradient materials, where the gradient could be not only in terms of mechanical properties or crosslinking, but also the extent of biological function that could be incorporated into these materials to emulate the biological environment. A large number of hydrogel materials are currently made using free radical polymerization, and this creates materials that are difficult to degrade or cannot degrade in vivo. Although degradation sequences have been implemented in these materials by means of degradable crosslinkers, the main chains still have the properties of polymers made by free radical polymerization. The latter limits, to some extent, the current use of these materials in many applications. A further need still exists to make hydrogel materials that degrade completely, and new research may offer solutions to this limitation in the future.
